# Fractalkine Induces Hepcidin Expression of BV-2 Microglia and Causes Iron Accumulation in SH-SY5Y Cells

**DOI:** 10.1007/s10571-019-00694-4

**Published:** 2019-06-06

**Authors:** Edina Pandur, Kitti Tamási, Ramóna Pap, Edit Varga, Attila Miseta, Katalin Sipos

**Affiliations:** 1 Department of Pharmaceutical Biology, Faculty of Pharmacy, University of Pécs, Rókus Str. 2, 7624 Pécs, Hungary; 2 Department of Laboratory Medicine, Medical School, University of Pécs, Ifjúság Str. 13, 7624 Pécs, Hungary

**Keywords:** Fractalkine, Hepcidin, Microglia, NFκB, TMPRSS6

## Abstract

Fractalkine (CX3CL1) is a potent inflammatory mediator of the central nervous system, which is expressed by neurons and regulates microglial functions by binding to fractalkine receptor (CX3CR1). It has been demonstrated that neuroinflammation plays an important role in iron accumulation of the brain leading to neuronal cell death. The major regulator of iron homeostasis is the peptide hormone hepcidin. Hepcidin expression is triggered by inflammatory conditions, which may contribute to the neuronal iron accumulation. In the present study, we established a bilaminar co-culture system of differentiated SH-SY5Y cells and BV-2 microglia as a neuronal model to examine the effect of soluble fractalkine on iron homeostasis of microglia and SH-SY5Y cells. We determined the hepcidin expression of fractalkine-treated microglia which showed significant elevation. We examined the relation between increased hepcidin secretion, the known hepcidin regulators and the signalling pathways controlled by fractalkine receptor. Our data revealed that TMPRSS6 and alpha 1-antitrypsin levels decreased due to fractalkine treatment, as well as the activity of NFκB pathway and the tyrosine phosphorylation of STAT5 factor. Moreover, fractalkine-induced hepcidin production of microglia initiated ferroportin internalisation of SH-SY5Y cells, which contributed to iron accumulation of neurons. Our results demonstrate that soluble form of fractalkine regulates hepcidin expression of BV-2 cells through fractalkine-mediated CX3CR1 internalisation. Moreover, fractalkine indirectly contributes to the iron accumulation of SH-SY5Y cells by activating ferroportin internalisation and by triggering the expressions of divalent metal transporter-1, ferritin heavy chain and mitochondrial ferritin.

## Introduction

Fractalkine or CX3CL1 belongs to the δ subfamily of chemokines (Harrison et al. 1998; Rostene and Buckingham 2007). It is a unique chemokine, which is constitutively expressed by neurons (Neiva et al. 2014). Fractalkine exists in two different forms: a membrane-bound glycoprotein providing direct interaction with its sole receptor, CX3CR1, which is found exclusively on microglia; and a soluble form produced by proteolytic cleavage of the N-terminal chemokine domain of the membrane-bound form working as an extracellular mediator (Cotter et al. 2002; Hundhausen et al. 2003; Clark et al. 2007; Sheridan and Murphy 2013). Fractalkine receptor is a Gi-protein coupled receptor; upon binding of membrane-bound fractalkine, it triggers several signal transduction pathways, like PI3K, AKT and NFκB regulating the different activities of microglia. Therefore, the fractalkine/CX3CR1 axis is involved in keeping microglia in resting state by regulating cytokine production. It is also involved in the control of microglia-mediated neurotoxicity and neuronal survival (Paolicelli et al. 2014; Lauro et al. 2015; Szepesi et al. 2018). Membrane-bound fractalkine also plays a part in neurogenesis and synaptic maturation, pruning, activity and plasticity (Paolicelli et al. 2011; Hoshiko et al. 2012; Ueno et al. 2013; Wolf et al. 2013). The soluble fractalkine also acts on CX3CR1 and triggers receptor internalisation and may provide anti-inflammatory and neuroprotective activities of microglia (Tarozzo et al. 2002; Morganti et al. 2012; Lee et al. 2014; Zhang et al. 2015). However, the exact role of fractalkine as a neuroprotective or a neurotoxic chemokine is still controversial (Mizuno et al. 2003; Lyons et al. 2009; Wynne et al. 2010; Lauro et al. 2015). Iron is an essential element required for many different biological processes: regulation of oxygen transport, cellular metabolism, DNA synthesis, growth and development, neurotransmission, myelination, and neuronal metabolism (Ward et al. 2014; Sangkhae and Nemeth 2017). A small peptide hormone, called hepcidin produced mainly by the liver, regulates the body iron homeostasis but it is also expressed in the cells of the CNS (Park et al. 2001; Zechel et al. 2006; Rishi et al. 2015). Hepcidin is transcribed as preprohepcidin, which, after translation, loses its leader sequence forming prohepcidin. Prohepcidin undergoes further cleavage by furin to form mature hepcidin (Valore and Ganz 2008). Hepcidin acts through its receptor, the only known iron exporter ferroportin. Upon hepcidin-binding ferroportin is internalised and degraded in the cell; thus, hepcidin decreases iron release and induces iron retention of the iron utilising or storing cells (Nemeth et al. 2004; Aguirre et al. 2005). Hepcidin expression is regulated by many extracellular signals such as hypoxia, iron availability and inflammation (Ganz 2003; Sonnweber et al. 2014; Schmidt 2015) via different signal transduction pathways (e.g. BMPR/BMP/HJV/SMAD, IL-6R/IL-6/JAK-STAT and MAPK/p38) (Wrighting and Andrews 2006; Babitt et al. 2006; Huang et al. 2009; Lu et al. 2015). The cooperation of many positive and negative transcriptional regulators maintains the balance of hepcidin synthesis (De Domenico et al. 2007; Muckenthaler 2008; Rishi et al. 2015).

The exact role of hepcidin in the brain is still unsolved. Astrocytes, microglia and neurons express hepcidin at different rate but the effect of hepcidin in the brain has not been fully described yet (Wang et al. 2010; Raha et al. 2013; Du et al. 2015). Astrocytes, neurons and microglia have the capacity to accumulate and store large quantities of iron (Bishop et al. 2011). Iron accumulation in the brain cells mediated by inflammation, neurodegeneration, brain injury or infections leads to oxidative damages, mitochondrial dysfunction and cell death (Núñez et al. 2012; Urrutia et al. 2014; Haider 2015). Meanwhile alterations in the iron metabolism are important triggering factors of neuroinflammation (Ke and Ming Qian 2003; Ong and Farooqui 2005; Hadzhieva et al. 2014; Li and Reichmann 2016).

Taken together the observations that fractalkine and iron metabolism both are affected by inflammation, we aimed to reveal the possible interactions between them. In the present study, we described a new regulatory pathway of hepcidin expression mediated by fractalkine in BV-2 microglial cells and investigated the effect of microglial hepcidin on the iron metabolism (iron uptake, storage and release) of differentiated SH-SY5Y cells using a bilaminar co-culture system.

## Methods

### Cell Cultures and Treatments

SH-SY5Y neuroblastoma cells (ATCC, CRL-2266) were cultured in Dulbecco’s Modified Eagle Medium/Nutrient Mixture F-12 medium (DMEM/F12; Lonza Ltd., Basel, Switzerland) supplemented with 10% fetal bovine serum (FBS, EuroClone S.p.A, Pero, Italy), 1% non-essential amino acids (NEAA, Lonza) and 1% penicillin–streptomycin (P/S, Lonza Ltd.). Cells (1 × 10^6^) were seeded onto Thermanox coverslips (Nunc, Thermo Fisher Scientific Inc., Waltham, MA, USA) in 6-well dishes (TPP Techno Plastic Products AG, Trasadingen, Switzerland) in DMEM/F12 medium supplemented with 1% FBS and 1% NEAA and were differentiated with 1 µM all-trans retinoic acid (ATRA, Sigma-Aldrich Kft, Budapest, Hungary) for 5 days. BV-2 murine microglial cells (kind gift from Prof. László Tretter and his research group) were maintained in Dulbecco’s Modified Eagle Medium (DMEM; Lonza Ltd.) supplemented with 10% FBS and 1% P/S. The cells were plated on poly-l-ornithine (Sigma-Aldrich Kft.)-coated dishes (Corning Inc., Corning, NY, USA). For the co-culture experiments, the differentiated SH-SY5Y cells were added to the BV-2 cells by turning the Thermanox coverslips upside down with SH-SY5Y cells facing to the microglial cells. The cells were separated by only a thin layer of culture medium. Bilaminar co-cultures were supplemented with 10 µM of cytosine-β-d-arabinofuranoside (Sigma-Aldrich Kft.), in order to prevent glia proliferation. Monocultures of SH-SY5Y and BV-2 cells and co-cultures were treated with the extracellular part of fractalkine (soluble fractalkine, 10 ng/ml) (PreproTech Inc., Rocky Hill, NJ, USA) for 6 h and 24 h to reveal the early and late changes of mRNA and protein expressions. Untreated cells (6 h and 24 h) were used as controls. All experiments were carried out in a humidified atmosphere containing 5% CO_2_ at 37 °C. Viability of the SH-SY5Y and BV-2 cells was measured using Cell-counting Kit-8 (CCK-8) cell viability assay (Sigma-Aldrich Kft.) after the separation of the two cell types in the treated co-cultures. The assay measures the dehydrogenase activity of the viable cells producing formazan from WST-8 reagent. Briefly, the co-culture was made in 24-well plate then after each treatment, the cells were separated. The coverslips containing the SH-SY5Y cells were transferred to an empty 24-well plate and then 400 µL medium was added to the wells. The BV-2 cells and SH-SY5Y cells were treated with 40 µL of tetrazolium salt WST-8 reagent and were incubated for 1 h at 37 °C and 5% CO_2_. After adding 40 µL 1% SDS, the absorbance of the samples was measured at 450 nm using EnSpire Multimode plate reader (PerkinElmer, Rodgau, Germany). Viability was expressed as percentile of the total cell number of the untreated control cells.

### Enzyme-Linked Immunosorbent Assay (ELISA) Measurements

After each treatment of the cells, culture media of the control and fractalkine-treated cells were collected and stored at − 80 °C until the measurements. The mature hepcidin content of each sample was determined with Mouse Hepcidin 25 ELISA Kit (Abbexa Ltd., Cambridge, UK) according to the manufacturer’s protocol. Secreted IL-6 concentrations of the culture media were determined with mouse IL-6 ELISA Kit (Thermo Fisher Scientific Inc.) according to the instructions of the manufacturer.

### Real-Time PCR

The co-cultured SH-SY5Y cells were separated from BV-2 cells by removing the coverslips from the surface of BV-2 cells. The coverslips were washed with phosphate-buffered saline (PBS; Sigma-Aldrich Kft.). The total RNA of SH-SY5Y cells was isolated directly from the coverslips. BV-2 cells were washed with PBS and then were harvested from the culture dishes after trypsinization. Total RNA was isolated from each sample using Quick RNA mini kit (Zymo Research, Irvine, CA, USA). Complementary DNA was synthesised from 200 ng total RNA using high capacity cDNA reverse transcription kit (Applied Biosystems, Thermo Fisher Scientific Inc.) according to the manufacturer’s protocol. Determination of gene expressions was performed in CFX96 Real-time System (Bio-Rad Inc. Hercules, CA, USA) using iTaq™ Universal SYBR^®^ Green Supermix (Bio-Rad Inc.). The total reaction volume was 20 μL, comprising 7.4 μL water, 0.4 μL of each primer (10 μmol/L), 10 μL Master Mix, and 20 ng (2 µL) of cDNA. The PCR amplification was created as follows: denaturation at 95 °C for 5 min, 45 PCR cycles of 95 °C for 12 s, 60 °C for 30 s, 72 °C for 30 s. Melting curves were generated after each quantitative PCR run to ensure that a single specific product was amplified. The melting curve was performed consisting of 30 s at 65 °C and slow heating at a rate of 0.5 °C per second to 95 °C with continuous fluorescence measurement (Pan et al. 2014, 2015). The relative quantification was calculated by the Livak (∆∆Ct) method using the Bio-Rad CFX Manager 3.1 software (Bio-Rad Inc.). The expression level of the gene of interest was compared with the level of β-actin in each sample. These relative expression rates were then compared between the treated and the untreated samples. The relative expression of the controls was regarded as 1. Controls were made at each examined time point of the treatments, 6 h and 24 h. The mRNA expression of the treated cells was compared to the appropriate controls. The primer sequences used in this study are described in Table 1.

**Table 1:** Real-time PCR gene primer list

Primer	Sequence 5′ → 3
BV-2	
β-actin forward	CTGTCGAGTCGCGTCCA
β-actin reverse	TCATCCATGGCGAACTGGTG
HAMP forward	GACATTGCGATACCAATGCAG
HAMP reverse	GCAACAGATACCACACTGGGA
TMPRSS6 forward	CTGTCCAACAGCTCAACCCT
TMPRSS6 reverse	TGTCGTTCACACTGGCTTCC
A1AT forward	TCTGGGACAGCAAGCTGAAA
A1AT reverse	GCTGGGGACTGATCCTTCTG
CX3CR1 forward	CCATTAGTCTGGGCGTCTGG
CX3CR1 reverse	GTCACCCAGACACTCGTTGT
IL-6 forward	CTCTGCAAGAGACTTCCATCCA
IL-6 reverse	GACAGGTCTGTTGGGAGTGG
IL-1β forward	TGCCACCTTTTGACAGTGATG
IL-1β reverse	TGATGTGCTGCTGCGAGATT
TNFα forward	GATCGGTCCCCAAAGGGATG
TNFα reverse	CCACTTGGTGGTTTGTGAGTG
SH-SY5Y	
β-actin forward	AGAAAATCTGGCACCACACC
β-actin reverse	GGGGTGTTGAAGGTCTCAAA
DMT-1 forward	GTGGTTACTGGGCTGCATCT
DMT-1 reverse	CCCACAGAGGAATTCTTCCT
FTH forward	GAGGTGGCCGAATCTTCCTTC
FTH reverse	TCAGTGGCCAGTTTGTGCAG
FTMT forward	AAGGTGACCCCCATTTGTGC
FTMT reverse	GGGGCCCCCATCTTCACTAA
CX3CL1 forward	TACCTGTAGCTTTGCTCATC
CX3CL1 reverse	GTCTCGTCTCCAAGATGATT

### Immunoblotting

SH-SY5Y cells were collected the same way as for the gene expression analysis. Pelleted cells were lysed with 150 µl of M-PER Mammalian Protein Extraction Reagent (Thermo Fisher Scientific Inc.) supplemented with Complete mini protease inhibitor cocktail (Roche Ltd., Basel, Switzerland). Protein contents of the samples were measured with DC Protein Assay Kit (Bio-Rad Inc.). The same amount of protein (15 µg) from each sample was loaded onto 12% or 14% SDS-PAGE and transferred by electro blotting to nitrocellulose membranes (Pall AG, Basel, Switzerland) (Pan et al. 2017). The membranes were blocked with 5% non-fat dry milk in TBST (Tris buffer saline, 0.1% Tween-20) for 1 h at room temperature. After the blocking step, the membranes were probed with the following polyclonal rabbit antibodies for 1 h at room temperature according to the manufacturer’s protocols: anti-FP IgG (1: 1000; Novus Biologicals, Biotechne R&D Systems Europe Ltd., Abingdon, UK), anti-DMT-1 IgG (1:1000; Novus Biologicals), anti-FTMT (1:500; Invitrogen, Thermo Fisher Scientific Inc.), anti-FTH (1:1000; Cell Signaling Technology Europe, Leiden, The Netherlands), anti-CX3CL1 (1:500; Invitrogen, Thermo Fisher Scientific Inc.) and anti-cleaved caspase-3 (1:1000; Cell Signaling Technology Europe). BV-2 cells were fractionated immediately after collection using Subcellular Protein Fractionation Kit for Cultured Cells (Thermo Fisher Scientific Inc.). The protein samples of each fraction were treated the same way as the SH-SY5Y protein samples. The following antibodies were used for the Western blot analyses according to the manufacturer’s protocols: anti-CX3CR1 IgG (1:1000; Sigma-Aldrich Kft.), TMPRSS6 IgG (1:1000; Sigma-Aldrich Kft.), A1AT IgG (1:1000; Abcam, Cambridge, UK) anti-Fp IgG (1:1000; Novus Biologicals, Biotechne R&D Systems Europe Ltd.), anti-NFκB1/p50 IgG (1:1000), anti-RELA IgG (1:2000), anti-p-STAT5 IgG (1:1000), anti-cleaved-caspase-3 (1:1000) and anti-p-STAT3 IgG (Cell Signaling Technology Europe). β-actin (1:2000; Sigma-Aldrich Kft.) was used as loading control in all Western blot experiments. Goat anti-rabbit (H + L) HRP-conjugate was used as secondary antibody (1:3000; Bio-Rad Inc.). Protein detection was carried out with WesternBright ECL chemiluminescent substrate (Advansta Inc., San Jose, CA, USA). Optical densities of Western blots were determined using ImageJ software (https://imagej.nih.gov/ij/) and were expressed as percentage of target protein/β-actin abundance.

### Determination of the Total Iron Content of Cultured Cells

Determination of the iron content was performed using a colorimetric ferrozine-based assay described by Riemer et al. (2004). Briefly, the SH-SY5Y cells and BV-2 cells were collected separately and lysed with 50 mM NaOH at room temperature for 2 h. After the incubation, the samples were mixed with iron releasing reagent (1.4 M HCl, 4.5% (w/v)) and were incubated for 2 h at 60 °C. After iron releasing from proteins, iron detection reagent (6.5 mM ferrozine, 6.5 mM neocuproine, 2.5 M ammonium acetate, 1 M ascorbic acid) was added to each tube and incubated at RT for 30 min. The absorbance was measured in 96-well dishes at 492 nm using Multiskan GO spectrophotometer (Thermo Fischer Scientific Inc.). The concentration was determined by a standard curve of FeCl_3_ (0-300 µM) treated the same way as the samples. Protein concentration was measured from each sample with DC Protein Assay Kit (Bio-Rad Inc.) and iron content was normalised against the protein content and was expressed as µM iron/mg protein. Iron contents of the untreated control cells were measured the same way and at the same time points of the experiments (6 h and 24 h).

### Statistical Analysis

The data presented are representative of at least three independent experiments. For all data, *n* corresponds to the number of independent experiments. Real-time PCR and cell viability assays were carried out in triplicate in each independent experiment. Statistical analysis was performed using SPSS software (IBM Corporation, Armonk, NY, USA). Statistical significance was determined using Student’s *t* test to compare treated groups (6 h and 24 h) to their appropriate control group (6 h and 24 h). We used Bonferroni correction to adjust probability values because of the increased risk of a type I error when making multiple statistical tests. Data are shown as mean ± standard errors of the mean (S.E.M.). Statistical significance was set at *p* value < 0.05.

## Results

### Fractalkine Induces Microglial IL-6 Production

**Fig. 1:**
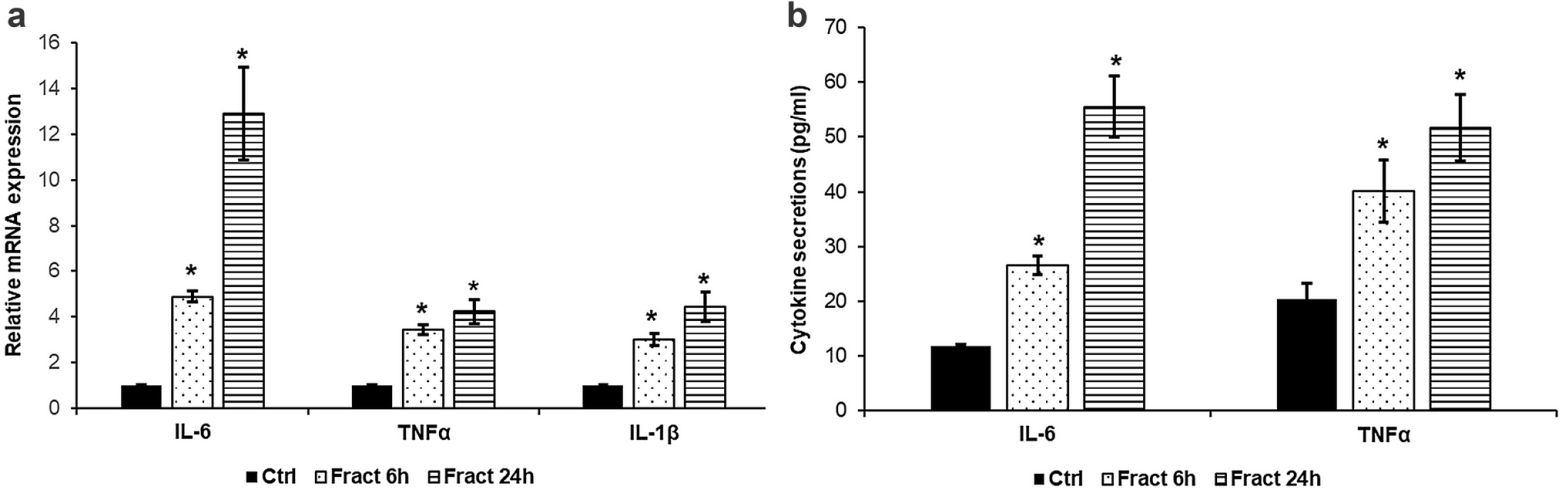
mRNA expression levels of IL-6, TNFα and IL-1β and concentrations of the secreted IL-6 and TNFα in fractalkine-treated co-cultured BV-2 cells. Real-time PCR was performed with SYBR green protocol using gene-specific primers. β-actin was used as housekeeping gene for the normalisation and relative expression of controls was regarded as 1. Secreted IL-6 and TNFα were measured with ELISA according to the manufacturer’s protocols. **a** Relative mRNA expression levels of IL-6, TNFα and IL-1β. **b** Concentrations of the secreted IL-6 and TNFα measured from the cell culture medium. The bars represent mean values and error bars represent standard errors of the mean (S.E.M.) for three independent experiments (*n* = 3). The asterisks mark *p* < 0.05 compared to the controls

Increased IL-6 as well as TNFα and IL-1β cytokine productions of the microglia are the major signs of their activation in response to inflammatory stimulus (e.g. LPS). We determined IL-6, TNFα and IL-1β mRNA expression levels of BV-2 cells and the concentrations of secreted IL-6 and TNFα proteins from the cell culture media after fractalkine (10 ng/ml) treatments of BV-2/SH-SY5Y co-cultures. IL-6 mRNA expressions of the examined cytokines were significantly elevated both after 6 h and 24 h treatments (Fig. 1a) and both IL-6 and TNFα protein secretions showed the same phenomenon (Fig. 1b) indicating the activation of microglia due to fractalkine and the interaction of the two cell types.

### Fractalkine Increases Hepcidin Secretion

**Fig. 2:**
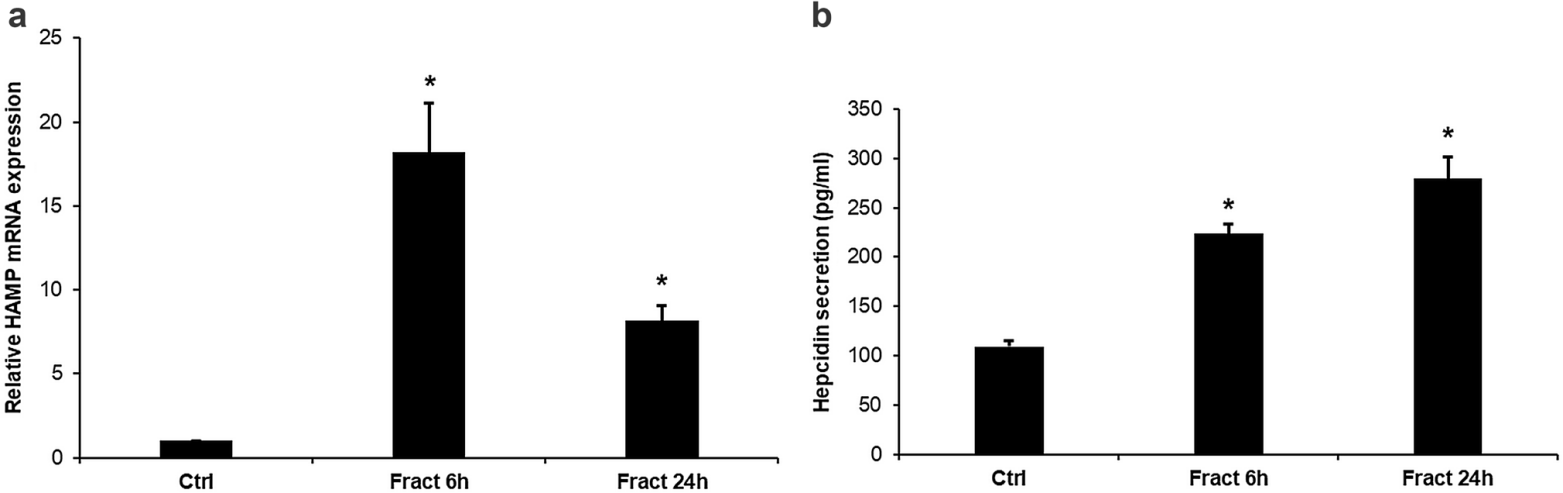
mRNA expression levels of HAMP and concentration of the secreted hepcidin in fractalkine-treated co-cultured BV-2 cells. Real-time PCR was performed with SYBR green protocol using gene specific primers. The β-actin was used as housekeeping gene for the normalisation and the relative expression of the controls was regarded as 1. Secreted hepcidin was quantified with ELISA according to the manufacturer’s instructions. **a** Relative mRNA levels of HAMP. **b** Concentration of the secreted hepcidin measured from the cell culture medium. The bars represent mean values and error bars represent standard errors of the mean (S.E.M.) for three independent experiments (*n* = 3). The asterisks indicate *p* < 0.05 compared to the controls

During inflammation, most of the hepcidin producing cells, especially macrophages, increase hepcidin secretion by activating cytokine receptors (e.g. IL-6 receptor). Since microglia can be considered as the macrophages of the central nervous system, we measured the hepcidin production of these cells to reveal whether fractalkine regulates hepcidin expression. Preprohepcidin mRNA (HAMP) expression was significantly elevated to 18.19 fold at 6 h and it decreased to 8.18 fold at 24 h long fractalkine treatment (Fig. 2a). Hepcidin productions of BV-2 cells determined from the co-culture media significantly increased compared to the control cells (Fig. 2b).

### Fractalkine Decreases the Expression Levels of the Hepcidin Regulators TMPRSS6 (Matriptase-2) and Alpha 1-Antirypsin

**Fig. 3:**
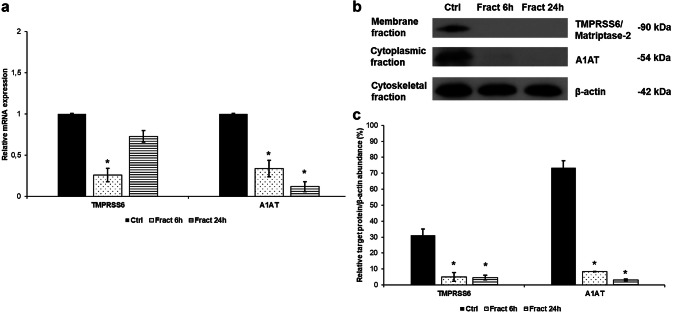
Analyses of the mRNA and protein levels of hepcidin regulators. Real-time PCR was performed with SYBR green protocol using gene specific primers. The β-actin was used as housekeeping gene for the normalisation and the relative expression of the controls was considered as 1. Pelleted BV-2 cells from the co-cultures were fractionated. Protein contents of the fractions were measured. The same amount of protein (15 µg) from each sample was loaded onto 12% SDS-PAGE and transferred by electroblotting to nitrocellulose membranes and was probed with TMPRSS6, A1AT and β-actin polyclonal rabbit antibodies according to the manufacturer’s protocols. **a** Relative mRNA expression levels of the negative hepcidin regulators TMPRSS6 and alpha-1 antitrypsin. **b** Protein levels of negative hepcidin regulators TMPRSS6 and A1AT. **c** Optical densities of Western blot analyses of TMPRSS6 and A1AT. The analyses were done using ImageJ software; the optical density of the examined proteins was expressed as percentage of target protein/β-actin abundance. The bars represent mean values and error bars represent standard errors of the mean (SEM) for three independent experiments (*n* = 3). The asterisks indicate *p *< 0.05 compared to the controls

Since fractalkine treatment increased hepcidin production of the microglia, we investigated the hepcidin regulators to identify (including A1AT, TMPRSS6, NFκB and IL-6/STAT3) which of them could contribute to this result. We found two negative regulators with decreased mRNA (Fig. 3a) and protein levels (Fig. 3b): TMPRSS6 is a regulator of mHJV cleavage and, thus, inhibitor of the BMP/SMAD signalling pathway. A1AT is an inhibitor of prohepcidin/hepcidin maturation. Since both of them are negative regulators, their downregulation can lead to increased hepcidin mRNA expression and a higher rate of prohepcidin/hepcidin conversion.

### Fractalkine Causes Internalisation of CX3CR1 Receptor and Downregulation of NFκB Signalling Pathway

Based on the previous results, we proposed a hypothesis on the effect of soluble fractalkine on CX3CR1 receptor under normal and inflammatory conditions and the subsequent changes in the regulation of TMPRSS6, A1AT and hepcidin expression. Under normal conditions, the neurons produce less soluble fractalkine than at inflammation, maintaining the activity of CX3CR1 and the subsequent cell signalling pathways (MAPK, NFκB, PLC) to regulate the activation of STAT5 and NFκB proteins. The active STAT5 and NFκB proteins are responsible for the normal expression rate of TMPRSS6 and A1AT to control HAMP mRNA expression and the maturation of prohepcidin to hepcidin.

**Fig. 4:**
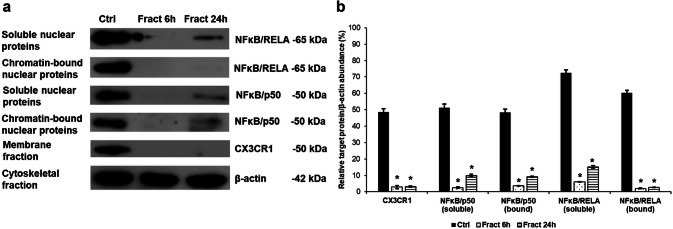
Western blot analysis of the proteins in the NFκB signalling pathways. Pelleted BV-2 cells from the co-cultures were fractionated and protein concentrations of the fractions were measured. The same amount of protein (15 µg) from each sample was loaded onto 12% SDS-PAGE and transferred by electro blotting to nitrocellulose membranes and were probed with CX3CR1, p50 (NFκB) and RELA (NFκB) polyclonal rabbit antibodies according to the manufacturer’s protocols. β-actin was used as loading control. **a** Protein levels of CX3CR1, p50 (NFκB) and RELA (NFκB) in the different protein fractions of BV-2 cells. **b** Optical densities of the Western blot analyses of CX3CR1, p50 (NFκB) and RELA (NFκB). The analyses were carried out using ImageJ software; the optical density of the examined proteins was expressed as percentage of target protein/β-actin abundance. The bars represent mean values and error bars represent standard errors of the mean (SEM) for three independent determinations (*n* = 3). The asterisks indicate *p* < 0.05 compared to the controls

The results of Western blot experiments confirmed our hypothesis that fractalkine has a role in the regulation of cell signalling pathways in microglia. Fractalkine induced the internalisation of its receptor CX3CR1 (Fig. 4), while the cell signalling pathways activated by CX3CR1 were downregulated. The soluble and chromatin-bound nuclear protein levels of p50 (NFκB) and RELA (NFκB) were decreased (Fig. 4) showing the decreased activity of NFκB pathway.

### Fractalkine Mediated CX3CR1 Internalisation Influences Tyrosine Phosphorylation of STAT Proteins

**Fig. 5:**
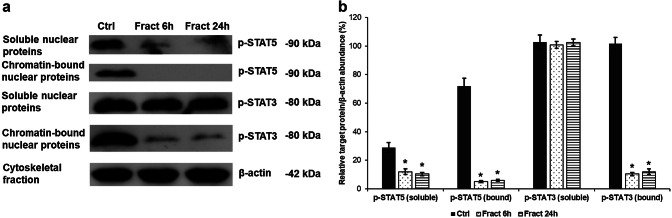
Western blot analysis of STAT5 and STAT3 tyrosine phosphorylation due to fractalkine treatment of co-cultured BV-2 cells. Pelleted BV-2 cells from the co-cultures were fractionated and protein concentrations of the fractions were measured. The same amount of protein (15 µg) from each sample was loaded onto 10% SDS-PAGE and transferred to nitrocellulose membranes and was probed with p-STAT5 or p-STAT3 polyclonal rabbit antibodies according to the manufacturer’s protocols. The β-actin was used as loading control. **a** Protein expression levels of p-STAT5 and p-STAT3 in the nuclear protein fractions of BV-2 cells. **b** Optical densities of the Western blot analyses of p-STAT5 and p-STAT3. The analyses were carried out using ImageJ software; the optical density of the examined proteins was expressed as percentage of target protein/β-actin abundance. The bars represent mean values and error bars represent standard errors of the mean (S.E.M.) for three independent experiments (*n* = 3). The asterisks indicate *p* < 0.05 compared to the controls

Tyrosine phosphorylation of STAT proteins is obligatory for their dimer formation and for binding to the regulatory DNA sequences of the target genes. Phosphorylation on specific serine amino acid residues of STATs by MAP kinases is important in the activation of transcription and seems to be essential for tyrosine phosphorylation as well (Decker and Kovarik 2000). Therefore, internalisation of CX3CR1 mediated by soluble fractalkine may influence tyrosine phosphorylation on STAT proteins through the downregulation of MAPK signalling pathway. Since both p-STAT5 and p-STAT3 can modify the rate of HAMP transcription (in a direct or an indirect way), we analysed the levels of p-STAT5 and p-STAT3 using Western blot technique. Fractalkine receptor internalisation decreased the tyrosine phosphorylation of STAT5 transcription factor both in the soluble protein fraction and in the chromatin-bound protein fraction (Fig. 5a, b) suggesting the importance of CX3CR1-regulated signalling pathways in the activation and in the binding of the transcription factor to the regulatory DNA sequences. Western blot analysis of p-STAT3 revealed that phosphotyrosine STAT3 protein levels were not changed in the soluble nuclear protein fraction but they were downregulated in the chromatin-bound nuclear protein fraction (Fig. 5a, b) suggesting the role of CX3CR1 internalisation in STAT3 phosphorylation. These results also indicate that STAT3 phosphorylation did not contribute to the elevated hepcidin expression in microglial cells.

### Microglial Hepcidin Internalises Ferroportin of the Differentiated SH-SY5Y Cells But Not of the BV-2 Cells

**Fig. 6:**
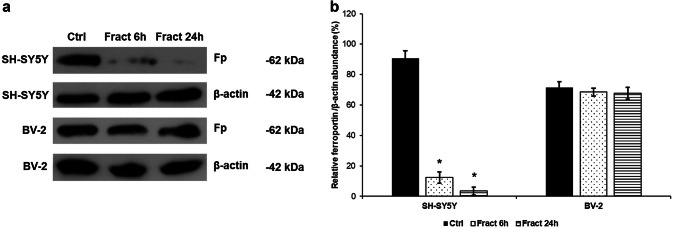
Western blot analysis of the iron exporter ferroportin (Fp) in BV-2 and differentiated SH-SY5Y cells after fractalkine treatment. SH-SY5Y cells were collected from Thermanox coverslips and BV-2 cells were collected from the cell culture dish with trypsinization. Cells were lysed and protein concentrations of the samples were measured. The same amount of protein (15 µg) from each sample was loaded onto 12% SDS-PAGE and transferred to nitrocellulose membranes and was probed with Fp polyclonal rabbit antibody according to the manufacturer’s protocol. The β-actin was used as loading control. **a** Protein expression levels of ferroportin in the BV-2 and SH-SY5Y cells. **b** Optical densities of the Western blot analyses of ferroportin. The analyses were carried out using ImageJ software; the optical density of the ferroportin was expressed as percentage of ferroportin protein/β-actin abundance. The bars represent mean values and error bars represent standard errors of the mean (S.E.M.) for three independent experiments (*n* = 3). The asterisks mark *p* < 0.05 compared to the controls

Hepcidin, the major regulator of iron metabolism acts by binding to its receptor ferroportin, the only known iron exporter. Hepcidin induces the internalisation of ferroportin, thus inhibiting iron release and causing iron retention in the cells. Since fractalkine induced hepcidin secretion of BV-2 cells, it was questionable whether hepcidin was acting on the producing BV-2 cells or on the SH-SY5Y cells. Western blot analysis of ferroportin revealed that microglial hepcidin caused receptor internalisation on the SH-SY5Y cells but not on the BV-2 cells (Fig. 6) suggesting that BV-2 cells could influence/regulate iron metabolism of SH-SY5Y cells by decreasing the iron export.

### SH-SY5Y Cells Increase Iron Uptake and Storage Capacities by Increasing the Expression of DMT-1, Ferritin and Mitochondrial Ferritin

**Fig. 7:**
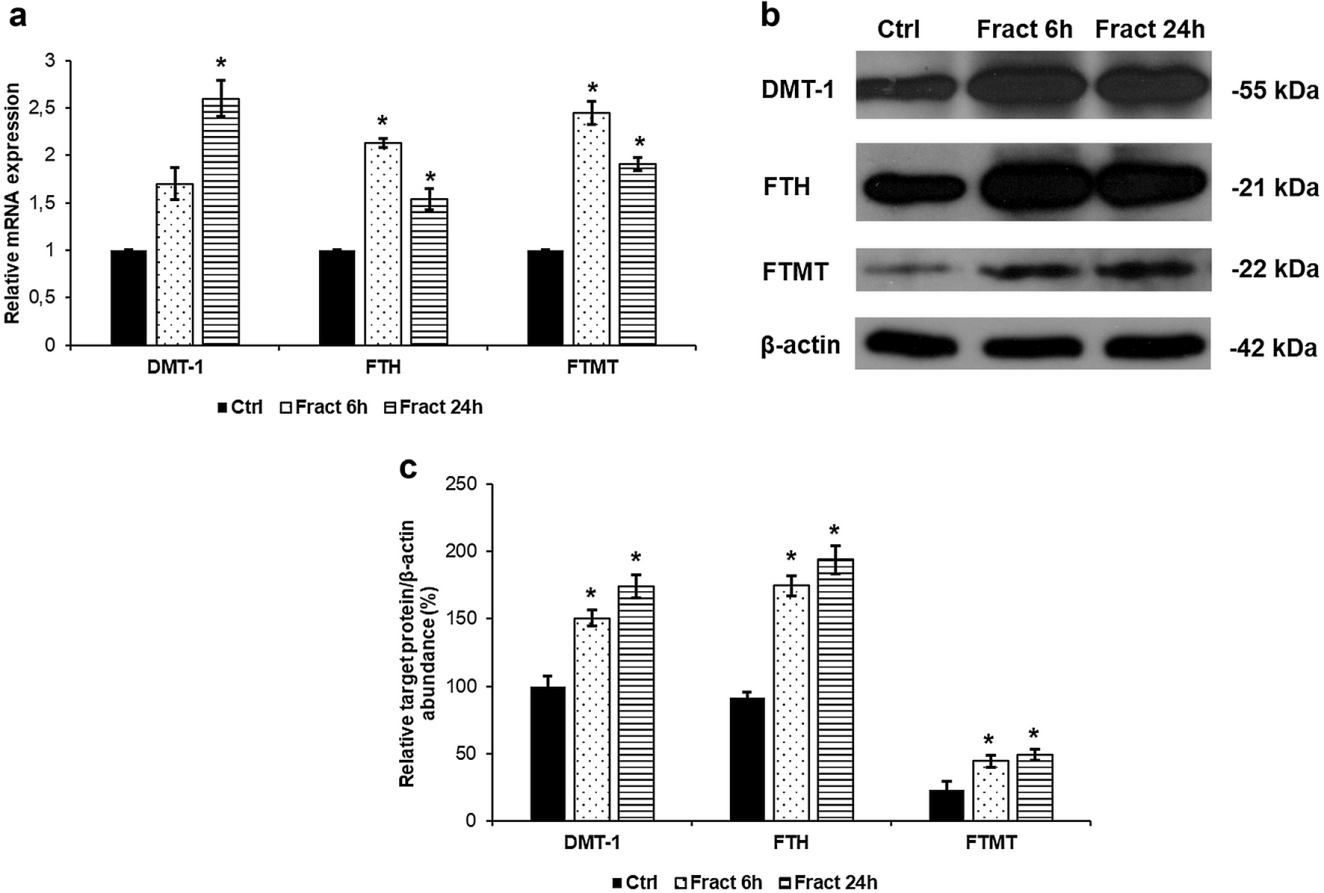
mRNA and Western blot analyses of genes responsible for iron uptake and storage in the co-cultured SH-SY5Y cells. Real-time PCR was performed with SYBR green protocol using gene-specific primers. The β-actin was used as housekeeping gene for the normalisation and the relative expression of the controls was considered as 1. Pelleted SH-SY5Y cells from the co-cultures were lysed and protein contents of the samples were measured. The same amount of protein (15 µg) from each sample was loaded onto 12% SDS-PAGE and transferred by electro blotting to nitrocellulose membranes and was probed with DMT-1, FTH and FTMT polyclonal rabbit antibodies according to the manufacturer’s protocols. The β-actin was used as loading control. **a** Relative mRNA expression levels of DMT-1, FTH and FTMT. **b** Protein levels of DMT-1, FTH and FTMT showing increased expressions. **c** Optical densities of the Western blot analyses of the DMT-1, FTH and FTMT. The analyses were carried out using ImageJ software; the optical densities of investigated proteins were expressed as percentage of target protein/β-actin abundance. The bars represent mean values and error bars represent standard errors of the mean for three independent experiments. The asterisks mark *p* < 0.05 compared to the controls

Our results revealed that BV-2 cells increased their hepcidin expression at fractalkine treatment and the consequential secreted microglial hepcidin decreased ferroportin expression of SH-SY5Y cells. Besides we investigated whether the presence of fractalkine-treated BV-2 cells in the co-culture influenced other iron proteins of SH-SY5Y cells, especially those that were responsible for iron transport and storage. The mRNA levels of the three examined genes divalent metal transporter-1 (DMT-1), ferritin heavy chain (FTH) and mitochondrial ferritin (FTMT) were elevated suggesting increased iron uptake into the cells, increased release of iron from the endosomes into the cytosol and elevated iron storage both in the cytosol and in the mitochondria (Fig. 7a). Western blot analysis showed elevated levels of the investigated proteins (Fig. 7b, c) proving the changes in iron metabolism of SH-SY5Y cells.

### Fractalkine Increases Iron Contents of BV-2 and SH-SY5Y Cells

**Fig. 8:**
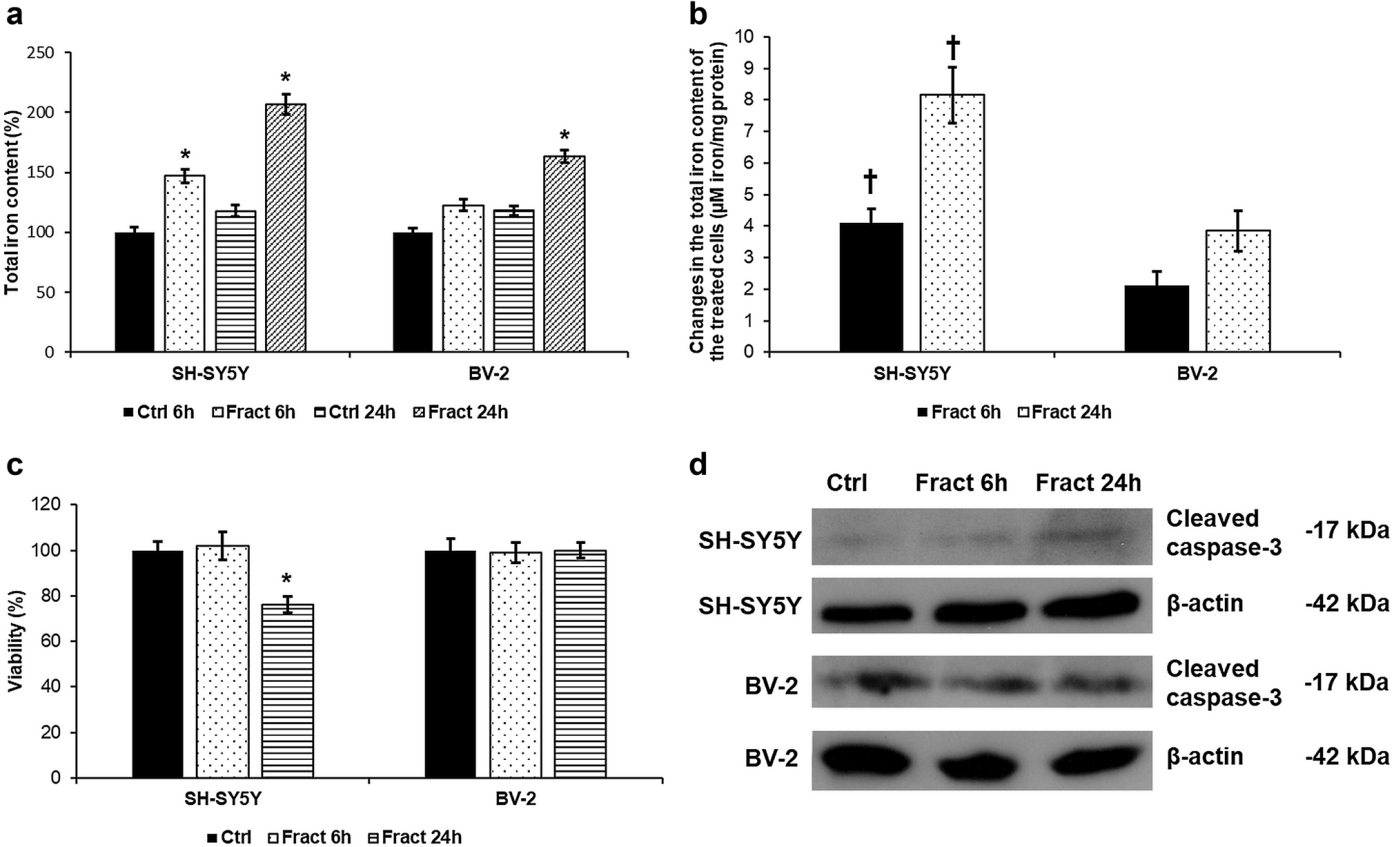
Iron measurements and cell viability determinations of the co-cultured SH-SY5Y and BV-2 cells. Intracellular iron contents of SH-SY5Y and BV-2 cells were determined using a colorimetric ferrozine-based assay and were expressed as % of the controls. Changes in the intracellular iron contents of the fractalkine-treated cells were expressed as µM iron/mg protein. The values are calculated from the differences between the treated and the control cells. Viability of the SH-SY5Y cells was measured using CCK-8 cell viability assay after the separation of the two cells types from the treated co-cultures. Viability is expressed as percentile of the total cell number of the untreated control cells. **a** Comparison of the total iron contents of fractalkine-treated, co-cultured SH-SY5Y and BV-2 cells. **b** Comparison of the changes of the total iron contents of fractalkine-treated, co-cultured SH-SY5Y and BV-2 cells. **c** Cell viability measurements of co-cultured SH-SY5Y and BV-2 cells after fractalkine treatment. **d** Protein levels of cleaved-caspase-3 in co-cultured SH-SY5Y and BV-2 cells. The bars represent mean values and error bars represent standard errors of the mean (S.E.M.) for four independent experiments (*n* = 4). The asterisks indicate *p* < 0.05 compared to the controls, while crosses mark difference *p* < 0.05 between the two cell types

The changes in the protein expression of iron uptake and storage genes suggest that SH-SY5Y cells increased their intracellular iron content. To prove our assumption, the iron content of both BV-2 and SH-SY5Y cells was measured using ferrozine-based spectrophotometric assay. The iron content of the control BV-2 cells was higher both at 6 h and 24 h (10.89 ± 0.86 and 12.77 ± 0.41 µM iron/mg protein, respectively) compared to the control SH-SY5Y cells (8.71 ± 1.16 and 10.25 ± 0.57 µM iron/mg protein) suggesting that at normal conditions microglia have a larger iron store that neurons. Fractalkine treatment increased the iron contents of both cell types but the total iron content of the SH-SY5Y cells was higher (147.18% at 6 h and 206.77% at 24 h) compared to the BV-2 cells (122.77% at 6 h and 163.17% at 24 h) when the control cells were consider as 100% (Fig. 8a). The changes in the total iron content of the fractalkine-treated cells indicated the significantly higher iron uptake and storage of SH-SY5Y cells (4.11 ± 0.45 µM iron/mg protein at 6 h and 8.16 ± 0.88 µM iron/mg protein at 24 h) compared to BV-2 cells (2.1 ± 0.43 µM iron/mg protein at 6 h and 3.85 ± 0.68 µM iron/mg protein at 24 h) (Fig. 8b). The increased iron content of the SH-SY5Y cells may contribute to the reduced cell viability. The living cell number of SH-SY5Y cells decreased at 24 h to 76% compared to the controls showing the effect of fractalkine and iron accumulation on the cell viability (Fig. 8c). We also examined the active caspase-3 protein expression in both SH-SY5Y cells and BV-2 cells to confirm the results of cell viability assay. Cleaved caspase-3 showed a minor elevation at 24 h in case of fractalkine-treated SH-SY5Y (Fig. 8d).

### The Presence of SH-SY5Y Cells is Obligatory to Maintain the Effect of Fractalkine on BV-2 Cells

**Fig. 9:**
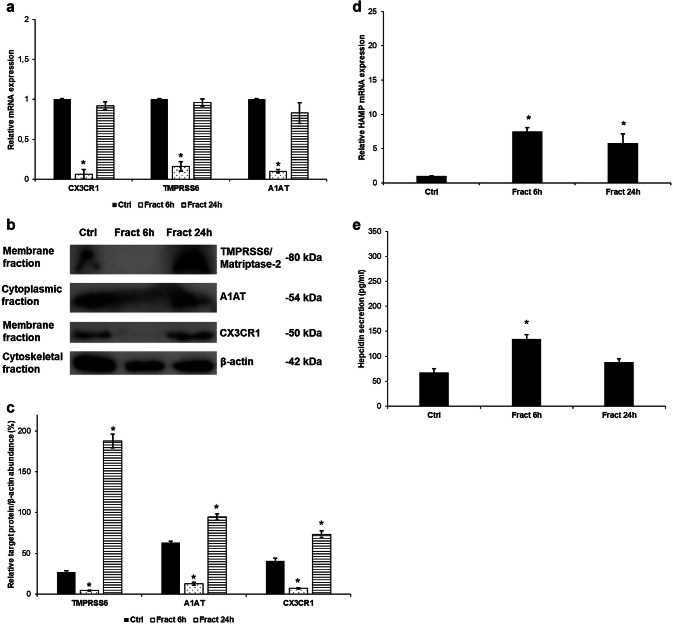
Effect of fractalkine on BV-2 monocultures. Real-time PCR was performed with SYBR green protocol using gene-specific primers. The β-actin was used as housekeeping gene for the normalisation and the relative expression of the controls was considered as 1. Pelleted BV-2 cells were fractionated and protein contents of the fractions were measured. The same amount of protein (15 µg) from each sample was loaded onto 12% SDS-PAGE and transferred to nitrocellulose membranes and was probed with CX3CR1, TMPRSS6 and A1AT polyclonal rabbit antibodies according to the manufacturer’s protocols. The β-actin was used as the loading control. Secreted hepcidin was quantified with ELISA kit according to the manufacturer’s instructions. **a** Relative mRNA expression levels of CX3CR1 and the negative hepcidin regulators TMPRSS6 and A1AT. **b** Protein levels of TMPRSS6, A1AT and CX3CR1. **c** Optical densities of the Western blot analyses of TMPRSS6, A1AT and CX3CR1. The analyses were done using ImageJ software; the optical density of the examined proteins was expressed as percentage of target protein/β-actin abundance. **d** Relative HAMP mRNA expression of the fractalkine-treated BV-2 cells. **e** Hepcidin secretion of the fractalkine-treated BV-2 cells. The bars represent mean values and error bars represent standard errors of the mean (SEM) for three independent experiments (*n* = 3 in each experiment). The asterisks indicate *p* < 0.05 compared to the controls

Fractalkine causes the activation of BV-2 cells and as a consequence of changing cell signalling pathways it increases the secretion of hepcidin. Although it is questionable whether the presence of SH-SY5Y cells is necessary for maintaining the downstream processes or BV-2 cells react the same way without the neurons in the presence of fractalkine alone. To find out the answer to this important question, we treated BV-2 cells with fractalkine the same way as in case of co-cultures and we determined the mRNA and protein expressions of the most important genes having a role in the regulation of hepcidin production of BV-2 cells. The mRNA expression analyses of CX3CR1, TMPRSS6 and A1AT revealed downregulation at 6 h but the levels returned to the control levels at 24 h (Fig. 9a). The same results were obtained by Western blot analysis. Protein levels of the investigated genes were downregulated at 6 h and then returned or showed increased expression compared to the control levels (Fig. 9b, c) suggesting the falloff of the effect of fractalkine on the BV-2 cells. HAMP mRNA expression was elevated at both time points (Fig. 9d), but these values were lower than in the co-cultured BV-2 cells. Hepcidin production increased compared to the untreated cells (Fig. 9e) but these values were also significantly lower compared to the co-cultured BV-2 cells: only 60% at 6 h and 31.5% at 24 h of the hepcidin production of co-cultured BV-2 cells indicating the reduced effect of fractalkine on BV-2 cells in the absence of SH-SY5Y cells.

**Fig. 10:**
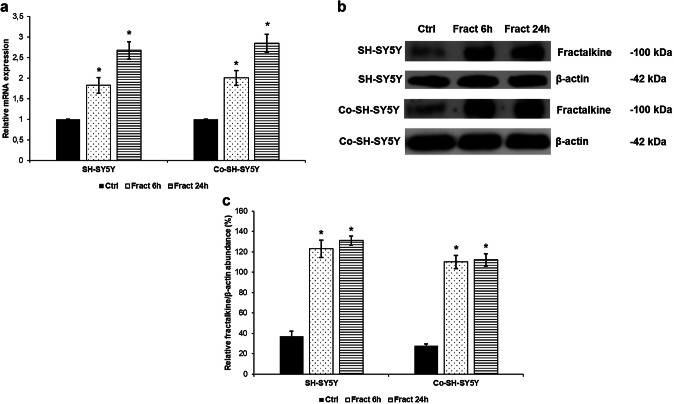
mRNA and Western blot analyses of fractalkine in the fractalkine-treated mono- and co-cultured SH-SY5Y cells. Real-time PCR was performed with SYBR green protocol using gene specific primers. The β-actin was used as housekeeping gene for the normalisation and the relative expression of the controls was considered as 1. Pelleted SH-SY5Y cells were lysed and protein contents of the samples were measured. The same amount of protein (15 µg) from each sample was loaded onto 10% SDS-PAGE and transferred by electro blotting to nitrocellulose membranes and was probed with fractalkine polyclonal rabbit antibodies according to the manufacturer’s protocol. The β-actin was used as loading control. **a** Relative mRNA expression levels of fractalkine in mono-and co-cultured SH-SY5Y cells. **b** Protein levels of fractalkine in fractalkine-treated SH-SY5Y cells. **c** Optical densities of the Western blot analysis of fractalkine. The analysis was carried out using ImageJ software, the optical density of the investigated protein was expressed as percentage of fractalkine protein/β-actin abundance. The bars represent mean values and error bars represent standard errors of the mean (SEM) for three independent experiments (*n* = 3). The asterisks mark *p* < 0.05 compared to the controls

Since not only neurons but also astrocytes are able to produce fractalkine in CNS, we examined whether fractalkine treatment of SH-SY5Y cells could trigger further fractalkine expression in neurons. The real-time PCR results revealed that SH-SY5Y cells increased their fractalkine mRNA expression both in mono- and co-cultures (Fig. 10a). The same results were obtained by Western blot experiments: the protein levels of fractalkine were elevated in the SH-SY5Y cells after fractalkine treatment (Fig. 10b). These findings may give the reason for the importance of SH-SY5Y cells in maintaining the effect of fractalkine on BV-2 cells in co-cultures.

## Discussion

Fractalkine regulates the interactions between neurons and microglial cells, the activation state of the microglia and, therefore, the inflammatory process of the brain, among other functions (Cotter et al. 2002; Hundhausen et al. 2003; Clark et al. 2007; Sheridan and Murphy 2013). Although the structure and function of fractalkine–fractalkine receptor axis are under strong investigations, the role of fractalkine in the brain is controversial (Lauro et al. 2015). Morganti et al. suggested that the soluble form of fractalkine triggered CX3CR1 internalisation and provided an anti-inflammatory and neuroprotective function in a mouse model of Parkinson’s disease (Morganti et al. 2012). According to the data obtained by Leonardi-Essmann et al., damaged neurons showed increased CX3CL1 secretion promoting phagocytic activity of microglia and activated anti-inflammatory and neuroprotective signals through the activation of JNK (Leonardi-Essmann et al. 2005). In spite of these observations, several publications deal with the neurotoxic effect of fractalkine in many models of chronic neurodegenerative disorders, e.g. Alzheimer’s disease and Parkinson’s disease (Liu et al. 2010; Shan et al. 2011).

Fractalkine is known to play a role in neuroinflammation. The activation of its receptor, CX3CR1, controls multiple cell signalling pathways, e.g. MAPK, NFκB and PI3K/AKT, and influences the production of pro-inflammatory cytokines (IL-6, IL-1β and TNFα) in microglia (Milligan et al. 2005; Ré and Przedborski 2006; Clark et al. 2007). Since inflammatory conditions are potent regulators of iron metabolism, which is also implicated in many neuronal diseases, we aimed to examine the possible role of fractalkine in the regulation of microglial and neuronal iron homeostasis.

The major regulator of the iron metabolism is the peptide hormone hepcidin that can decrease iron release from the cells via its receptor ferroportin (Nemeth et al. 2004) and, as a consequence, it triggers iron accumulation. Hepcidin expression is regulated by many extracellular signals. The iron deficiency and the pro-inflammatory cytokines/inflammation (IL-6, IL-1β and TNFα) act as positive regulators (Vela 2018a). Hypoxia and erythropoiesis (erythropoietin, TSWG1, GDF15 transcription factors and erythroferrone) act as negative regulators of hepcidin expression (Kautz et al. 2014; Arezes et al. 2018; Ganz, 2018). Moreover, many other regulatory proteins act on the hepcidin secreting cells, e.g. TMPRSS6-sHJV/mHJV-BMP/BMPR-SMAD complex, furin and A1AT. These regulators have a role in altering hepcidin expression both at transcriptional and translational levels (Pandur et al. 2009; Rishi et al. 2015; Sangkhae and Nemeth 2017).

Our previous results revealed that neuroinflammation triggered by bacterial cell wall components altered neuronal iron metabolism and caused iron accumulation in SH-SY5Y cells (Pandur et al. 2019). It has been revealed that neuronal fractalkine production is enhanced during brain injury and inflammatory conditions (Lauro et al. 2015). We established a bilaminar co-culture system of BV-2 microglia and differentiated SH-SY5Y cells to determine whether fractalkine is a potential regulator of iron homeostasis. In our co-culture system, the two cell types are able to contact with each other physically, by secreting cytokines and other mediators (Bureau et al. 2008; Cook et al. 2010) and they react to these stimuli. Moreover, using the co-culture model both the cytokine productions and the intracellular changes can be examined separately at the same time.

After fractalkine treatment, the microglial cells increased their IL-6, TNFα and IL-1β expressions suggesting fractalkine-induced microglial activation. Fractalkine-treated microglial cells showed elevated hepcidin secretion suggesting that soluble CX3CL1 influences microglial hepcidin synthesis. Since IL-6 is a potent activator of hepcidin expression by activating the JAK/STAT3 signalling pathway (Huang et al. 2005), STAT3 tyrosine phosphorylation was examined by Western blot analysis. The amount of p-STAT3, which is the most powerful positive regulator of hepcidin expression under inflammatory conditions, was not elevated in BV-2 cells during fractalkine treatment. This increases the possibility that the changes in the expression of the negative regulators are responsible for the altered hepcidin synthesis. Therefore, we focused on the negative regulators of hepcidin synthesis which could be affected by the signalling pathways controlled by fractalkine–fractalkine receptor interaction in microglia.

Meynard and their colleagues proved that TMPRSS6, one of the most important negative regulators of hepcidin transcription, is downregulated by inflammation via decreasing the phosphorylation rate of STAT5 (Meynard et al. 2013). We examined TMPRSS6 expression both at transcriptional and translational levels and we found that the expression levels significantly decreased in BV-2 cells at fractalkine treatment. We also determined tyrosine phosphorylation of STAT5 which showed decreased levels both in the soluble and chromatin-bound nuclear protein fractions. The alteration of TMPRSS6 protein level may contribute to the elevated HAMP expression. Interestingly, STAT3 phosphorylation only decreased in the active, chromatin-bound protein fraction suggesting that serine phosphorylation of STAT factors may be also affected by fractalkine. It has been proved that serine phosphorylation of STAT3 contributes to its maximal transcriptional activity (Wen and Darnell 1997; Shen et al. 2004) and may be necessary for DNA binding (Decker and Kovarik 2000). These data suggest that crosstalk between MAP kinases and JAK/STAT pathways is required to induce transcription of the target genes (Korzus et al. 1997). Since CX3CR1 was internalised by fractalkine treatment in the BV-2 cells, we analysed the effect of receptor internalisation on the NFκB pathway which may contribute to hepcidin upregulation. Both p50 and RELA protein levels were downregulated after fractalkine treatment suggesting the decreased activity of the signalling pathway.

**Fig. 11:**
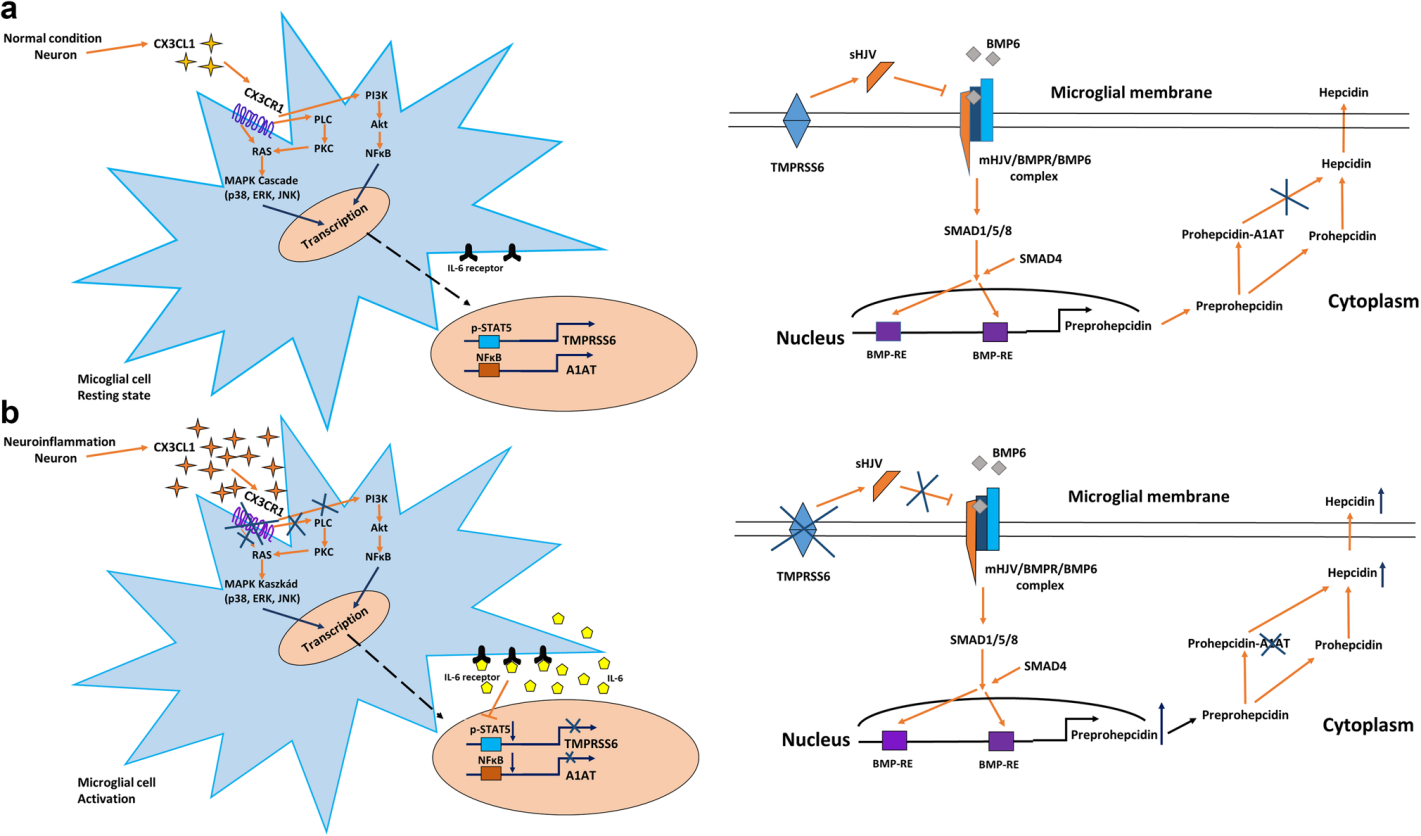
Regulation of HAMP mRNA expression and hepcidin maturation/secretion in microglia through activation/internalisation of CX3CR1 receptor at **a** normal and **b** inflammatory conditions. The key molecule of hepcidin regulation is CX3CR1 which controls cell signalling pathways that play a role in the transcriptional regulation of the two negative hepcidin regulators TMPRSS6 and A1AT. Internalisation of the receptor will cause the downregulation of TMPRSS6 and A1AT mRNA expressions and as a consequence HAMP transcriptional rate will decrease. Decreasing level of A1AT enhances prohepcidin–hepcidin conversion. For further explanation, see discussion section

Based on our results, we proposed a possible regulatory mechanism of soluble fractalkine on CX3CR1 receptor under normal and inflammatory conditions. Under normal conditions, the neurons produce less soluble fractalkine than at inflammation, maintaining the activity of CX3CR1 and the subsequent cell signalling pathways (MAPK, NFκB, PLC) to regulate the activation of STAT5 and NFκB proteins. The active STAT5 and NFκB proteins are responsible for the normal expression rate of TMPRSS6 and A1AT to control HAMP mRNA expression and the maturation of prohepcidin to hepcidin. TMPRSS6 membrane protein can cleave the membrane-bound hemojuvelin (mHJV) to a soluble form (Ramsay et al. 2009). Soluble hemojuvelin (sHJV) competes with mHJV and inhibits the formation of the active mHJV/BMPR/BMP complex (Finberg et al. 2010). Soluble hemojuvelin is responsible for regulating the transcription rate of HAMP through the BMP/SMAD signalling pathway (Fig. 11a). A1AT is serine protease inhibitor that can bind to prohepcidin in the cells (Pandur et al. 2019), inhibiting the conversion of prohepcidin to hepcidin. This way A1AT can regulate the amount of mature hepcidin produced in the cells (Fig. 11b).

Inflammatory conditions, which increase the neuronal fractalkine production, consequently cause the internalisation of CX3CR1 and terminate the aforementioned signalling cascades. The decreasing levels of active STAT5 and NFκB imply the downregulation of TMPRSS6 (Meynard et al. 2013) and A1AT (Ray et al. 1995; Pastore et al. 2013) transcription rates (Fig. 11b). Moreover, activation of the microglia increases IL-6 secretion that can also contribute to the decreasing STAT5 phosphorylation level (Meynard et al. 2013). Consequently, the amount of TMPRSS6 protein reduces in the plasma membrane decreasing the level of sHJV and causing the activation BMP/SMAD signalling pathway. The activated pathway induces preprohepcidin transcription and increases the preprohepcidin mRNA level which leads to increased intracellular prohepcidin protein level. On the other hand, reduced level of A1AT enhances prohepcidin cleavage and increases hepcidin concentration (Fig. 11b).

**Fig. 12:**
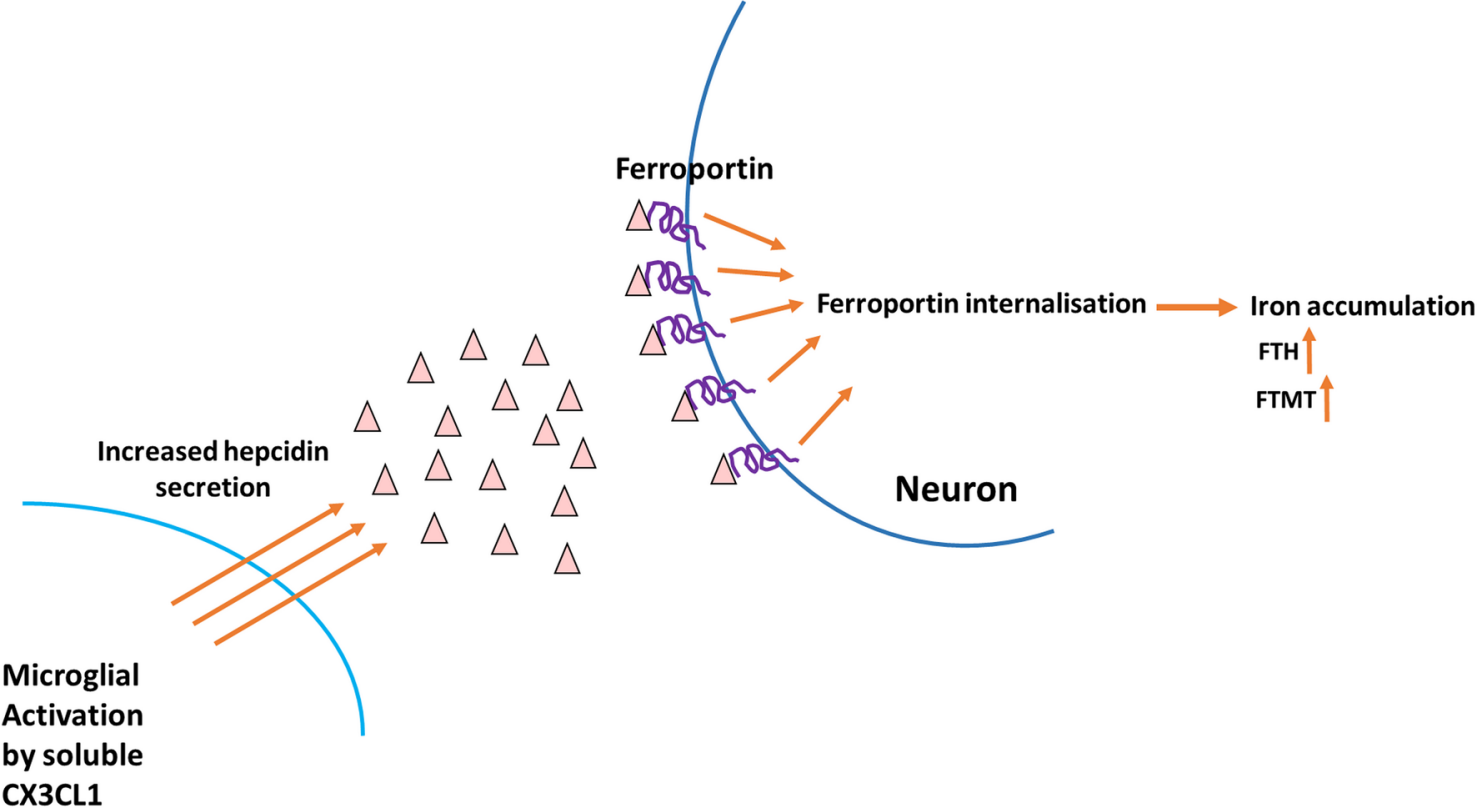
Effect of microglial hepcidin on differentiated SH-SY5Y cells. Secreted microglial hepcidin binds to its cognate receptor ferroportin expressed by SH-SY5Y cells. Upon hepcidin binding ferroportin is internalised reducing iron release and increasing iron retention. FTH and FTMT expression levels increased, suggesting iron accumulation

The major function of hepcidin in the brain is to decrease iron export from cells by inducing the internalisation of the iron exporter ferroportin which leads to intracellular iron retention (Vela 2018b). We examined whether increased microglial hepcidin secretion has an effect on ferroportin of BV-2 and SH-SY5Y cells. Interestingly, hepcidin caused ferroportin internalisation only on SH-SY5Y cells suggesting that microglial hepcidin act in a non-autocrine way. DMT-1 an iron importer is also affected by neuroinflammation (Urrutia et al. 2017; Vela 2018a) which is parallel with our results: neuronal ferroportin internalisation is followed by intracellular iron retention which is fortified by increased DMT-1 iron importer protein level. Cytosolic ferritin heavy chain (FTH) and mitochondrial ferritin (FTMT) expressions were also elevated both at mRNA and protein levels presuming iron accumulation in SH-SY5Y cells. The iron content measurements verify iron accumulation since total iron content of SH-SY5Y cells significantly increased parallel with hepcidin mediated ferroportin internalisation (Fig. 12). Although FTMT is supposed to provide protection for the cells from iron mediated damages by decreasing cytosolic iron content (Nie et al. 2005), overexpression of FTMT can increase the rate of apoptosis as well (Santambrogio et al. 2011). Our results revealed that iron accumulation decreased the viability of SH-SY5Y cells.

We revealed that BV-2 cells increased their TNFα and IL-1β cytokine expression levels due to fractalkine treatment. Microglial TNFα and IL-1β may also contribute to the iron accumulation of SH-SY5Y cells since these cytokines are able to increase the iron influx and decrease iron release of neurons by influencing the mRNA expression levels of DMT-1 and ferroportin (Wang et al. 2013; Urrutia et al. 2013).

The role of microglia in the dysregulation of neuronal iron metabolism is intensively investigated. The role of hepcidin in the brain iron metabolism is still controversial. It has been revealed that microglia contribute to the changes of iron homeostasis in neurons and they contribute to neuronal cell death at neuroinflammation.

In summary, our study describes a new hepcidin regulatory pathway in BV-2 microglial cells regulated by the fractalkine (CX3CL1)—fractalkine receptor (CX3CR1) axis. Fractalkine influences hepcidin expression both at transcriptional and translational levels. It decreases the activity of STAT5 transcription factor by reducing phosphorylation rate. Whether it is a direct or indirect effect is remained to be elucidated. Fractalkine decreased the transcriptional rate of TMPRSS6, a negative regulator of hepcidin transcription. Fractalkine also reduced the activity of NFκB signalling pathway and the transcription of A1AT, an inhibitor of hepcidin maturation. The alterations in the expressions of the aforementioned proteins resulted in an increased hepcidin transcription and an enhanced hepcidin secretion of BV-2 cells. Microglial hepcidin induces ferroportin internalisation and causes iron overload in SH-SY5Y cell decreasing their viability. Taken together we suggest that fractalkine–fractalkine receptor interaction is an important regulator of hepcidin and brain iron metabolism and microglia are crucial in maintaining neuronal iron homeostasis.
